# A SARS-CoV-2 mutant from B.1.258 lineage with ∆H69/∆V70 deletion in the Spike protein circulating in Central Europe in the fall 2020

**DOI:** 10.1007/s11262-021-01866-5

**Published:** 2021-08-27

**Authors:** Broňa Brejová, Kristína Boršová, Viktória Hodorová, Viktória Čabanová, Lenka Reizigová, Evan D. Paul, Pavol Čekan, Boris Klempa, Jozef Nosek, Tomáš Vinař

**Affiliations:** 1 Faculty of Mathematics, Physics and Informatics, Comenius University in Bratislava, Mlynská dolina, 842 48 Bratislava, Slovak Republic; 2 Institute of Virology, Biomedical Research Center of the Slovak Academy of Sciences, Dúbravská cesta 9, 845 05 Bratislava, Slovak Republic; 3 Department of Microbiology and Virology, Faculty of Natural Sciences, Comenius University in Bratislava, Ilkovičova 6, 842 15 Bratislava, Slovak Republic; 4 Department of Biochemistry, Faculty of Natural Sciences, Comenius University in Bratislava, Ilkovičova 6, 842 15 Bratislava, Slovak Republic; 5 Regional Authority of Public Health, Trenčín, Slovak Republic; 6 Department of Laboratory Medicine, Faculty of Healthcare and Social Work, Trnava University, Trnava, Slovak Republic; 7 MultiplexDX, s.r.o., Comenius University in Bratislava Science Park, Ilkovičova 8, 841 04 Bratislava, Slovak Republic

**Keywords:** SARS-CoV-2, B.1.1.7, B.1.258, Variant, Spike, Deletion

## Abstract

SARS-CoV-2 mutants carrying the ∆H69/∆V70 deletion in the amino-terminal domain of the Spike protein emerged independently in at least six lineages of the virus (namely, B.1.1.7, B.1.1.298, B.1.160, B.1.177, B.1.258, B.1.375). We analyzed SARS-CoV-2 samples collected from various regions of Slovakia between November and December 2020 that were presumed to contain B.1.1.7 variant due to drop-out of the Spike gene target in an RT-qPCR test caused by this deletion. Sequencing of these samples revealed that although in some cases the samples were indeed confirmed as B.1.1.7, a substantial fraction of samples contained another ∆H69/∆V70 carrying mutant belonging to the lineage B.1.258, which has been circulating in Central Europe since August 2020, long before the import of B.1.1.7. Phylogenetic analysis shows that the early sublineage of B.1.258 acquired the N439K substitution in the receptor-binding domain (RBD) of the Spike protein and, later on, also the deletion ∆H69/∆V70 in the Spike N-terminal domain (NTD). This variant was particularly common in several European countries including the Czech Republic and Slovakia but has been quickly replaced by B.1.1.7 early in 2021.

**Supplementary Information:**

The online version contains supplementary material available at 10.1007/s11262-021-01866-5.

The SARS-CoV-2 mutants carrying the Spike ∆H69/∆V70 deletion are easily misidentified in routine RT-qPCR as quickly spreading variant B.1.1.7. Here, we demonstrate that many Slovak samples collected in December 2020, originally presumed to contain B.1.1.7, belong to a sublineage of B.1.258, which acquired the Spike N439K substitution followed by the ∆H69/∆V70 deletion. Here, we denote this sublineage as B.1.258 ∆H69/∆V70 to distinguish it from sublineages without the ∆H69/∆V70 deletion (most samples in sublineages B.1.258.1–B.1.258.3 and B.1.258.14–B.1.258.16).

The variant has been highly prevalent in the Czech Republic (~ 59% sequenced samples between September and December 2020), Slovakia (~ 25% sequenced samples over the same period), and several other countries. The ∆H69/∆V70 deletion is associated with increased infectivity and evasion of the immune response [1] and evidence suggests that this mutation has arisen in B.1.258 independently of the B.1.1.7 variant. The deletion is likely to cause a drop-out of the Spike gene target in some RT-qPCR assays [2–5] and thus its carriers can be easily misidentified as B.1.1.7 (as happened in Slovakia [6]). The B.1.258 ∆H69/∆V70 variant also contains the N439K mutation in the receptor-binding domain (RBD) of the Spike protein, enhancing its affinity to the ACE2 receptor and facilitating escape from monoclonal antibodies and convalescent sera [7]. The lineage B.1.258 is generally characterized by five mutations (one in ORF1a, two in ORF1b, and two in Spike genes) but can be further divided into 21 sublineages differing by various combinations of additional 40 mutations. The ∆H69/∆V70 deletion is listed as one of the characteristic mutations for 14 of these sublineages (B.1.258.4–7, 9, 11, 12, 17–23) [8]. Most of the B.1.258 ∆H69/∆V70 samples also include substitutions in the virus replication proteins, namely, NSP9 M101I, NSP12 V720I, and NSP13 A598S.

The ∆H69/∆V70 deletion has arisen independently at least six times (Fig. 1A), frequently co-occurring with mutations in the Spike receptor-binding domain (RBD) such as N439K, Y453F, and N501Y [9–13]. Besides the B.1.258 ∆H69/∆V70 and B.1.1.7 variants, the deletion has been observed in B.1.1.298 (Danish mink farm outbreak [10, 14]) and B.1.375 (USA [15, 16]). Recurrent emergence even within well-established clades (such as EU1 (B.1.177) and EU2 (B.1.160)) suggests that ∆H69/∆V70 can increase overall fitness in concert with mutations that would be otherwise neutral or lower the infectivity [7, 9].

**Fig. 1:**
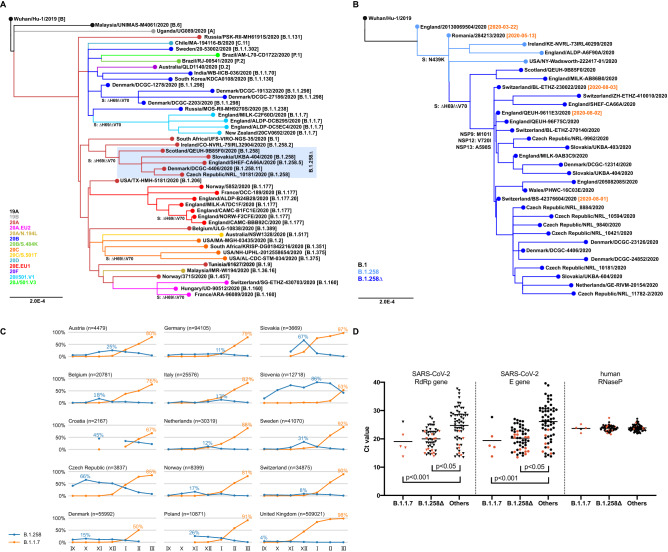
**A** Points of recurrent emergence of the ∆H69/∆V70 mutation. Nextclade lineages [20] in color. Pangolin lineages [12] in brackets. B.1.258∆ denotes B.1.258 with ∆H69/∆V70 deletion. **B** Origins of B.1.258 ∆H69/∆V70 variant. Mutations S:N439K, S:∆H69/∆V70, NSP9:M101I, NSP12:V720I, and NSP13:A598S are marked. Collection dates near to the important branching points are shown. The samples shown in the phylogenetic trees were selected from GISAID database [20] to cover significant lineages of interest (B.1.258 ∆H69/∆V70 variant in different countries and its outgroups and lineages containing ∆H69/∆V70 mutation and their outgroups). Phylogenetic trees were built using Augur v. 6 [21]. The prevalence was assessed based on all samples in GISAID (downloaded on April 29, 2021) for a particular country based on PANGO lineage classification [12] provided in GISAID metadata. **C** Prevalence of B.1.258 and B.1.1.7 variants in selected countries out of GISAID samples collected between September 2020 and March 2021. The highest monthly prevalence of both B.1.258 and B.1.1.7 is shown for each country. B.1.258 counts include all sublineages, regardless of the presence of ∆H69/∆V70 mutation. Only months where at least 20 samples were sequenced in the country are shown. Note that the samples may not be representative, as the sampling strategy differs from country to country, and it also changes over time. **D** Ct values in the swab specimens from the city of Trenčín (Slovakia) mass testing grouped according to the identified lineages. Ct values from routine RT-qPCR assay targeting RdRp, E, and human RNase P genes (used as a control to exclude possible impact of the sample quality) are shown. Classification of samples marked in red was confirmed by sequencing. B.1.258∆ denotes B.1.258 with ∆H69/∆V70 deletion. RT-qPCR assays were performed on RNA extracted by the Biomek i5 Automated Workstation using the RNAdvance Viral kit (Beckman Coulter, Indianapolis, Indiana, USA) from swab samples previously collected for the primary diagnostics. Besides rTEST COVID-19 RT-qPCR Allplex kit (MultiplexDX, Bratislava, Slovakia) targeting the RNA-dependent RNA polymerase (RdRp) and Envelope (E) genes, the newly developed rTEST COVID-19 qPCR B.1.1.7 kit (MultiplexDX, Bratislava, Slovakia) was used to differentiate B.1.1.7 and B.1.258 ∆H69/∆V70 variants [17]. The real-time PCR was performed on a QuantStudio™ 5 Real-Time PCR System (Applied Biosystems, Foster City, California, USA). The SARS-CoV-2 sequences were determined on a MinION sequencer (Oxford Nanopore Technologies) using a protocol based on PCR-tiling of 2-kb long amplicons [22]. The horizontal lines represent mean values. The differences in Ct values were statistically evaluated by unpaired *t* test using GraphPad Prism version 8.4.0

The earliest B.1.258 samples with ∆H69/∆V70 deletion were observed in Switzerland and in the UK at the beginning of August 2020 (Fig. 1B). The Spike N439K mutation has emerged before the ∆H69/∆V70 deletion (see sample from Romania as early as May 13, 2020); the outgroup that does not contain N439K has been observed in England on March 22, 2020. During the fall of 2020, B.1.258 variant gained significant prevalence in multiple countries, including Croatia, the Czech Republic, Sweden, Slovakia, Slovenia, Poland, Denmark, and Austria (Fig. 1C). Analysis of disease incidence and hospitalization data did not reveal any clear trends that would be common to all or most of the examined countries when correlated with B.1.258 prevalence. Nevertheless, in several countries (e.g., Austria, the Czech Republic, and Slovakia), the increasing incidence and hospitalization trends started already during the fall of 2020 while the B.1.1.7 variant dominance started later, early in 2021. One can therefore speculate that worsening of the epidemiologic situation in those countries could be at least partially attributed to the spread of the B.1.258 variant.

A newly developed RT-qPCR assay differentiating B.1.1.7 from other ∆H69/∆V70 variants [17] indicated that out of 122 clinical samples from a mass testing campaign in the city of Trenčín (Slovakia) in December 2020, only 5 (4.1%) belonged to the B.1.1.7 lineage, while an additional 50 (41.0%) samples carried the ∆H69/∆V70 deletion but were not B.1.1.7 (selected samples were later confirmed as B.1.258 ∆H69/∆V70 by sequencing). A routine RT-qPCR assay showed significantly lower Ct values in the swab samples for both B.1.1.7 and B.1.258 ∆H69/∆V70 samples, reflecting higher viral loads in patients carrying these variants (Fig. 1D).

Altogether, we have described a B.1.258 ∆H69/∆V70 variant of the SARS-CoV-2 virus that contains the S:N439K mutation shown to enhance the binding affinity of the Spike protein to human ACE2 receptor and facilitating escape from immune response, the S:∆H69/∆V70 mutation, which is known to increase viral infectivity, as well as several other non-synonymous mutations in NSP9, NSP12, and NSP13 likely affecting viral replication. RT-qPCR analysis on random samples collected during a mass testing campaign indicates that B.1.258 ∆H69/∆V70 samples carry higher viral loads compared to other strains, similarly as in the case of the B.1.1.7 variant. Interestingly, the B.1.258.17 sublineage has accumulated a higher number of mutations compared to other B.1.258 samples, including additional substitutions in the Spike protein (L189F, V772I), helicase NSP13 (P53L), and a substitution Q185H in ORF3a involved in apoptosis [18].

B.1.258 ∆H69/∆V70 variant shares many characteristics with quickly spreading B.1.1.7 and is likely responsible for worsening of the epidemiological situation in several countries, including Slovakia and the Czech Republic, in the fall of 2020. While in Slovenia the B.1.258 variant was still one of the major strains as of March 2021, in Slovakia the B.1.1.7 variant has extremely quickly replaced the B.1.258 by the beginning of February 2021 (estimate of ~ 74% of B.1.1.7 and ~ 6% of B.1.258 nationwide on February 3, 2021 by differential RT-qPCR tests [19] compared to 4.1% of B.1.1.7 and 41% of B.1.258 in the city of Trenčín on December 19–20, 2020). The B.1.258 ∆H69/∆V70 variant, as most of any other variants, was quickly replaced by B.1.1.7 variant early in 2021. Most recently, isolated cases associated with B.1.258 ∆H69/∆V70 variant were reported from Slovenia and Croatia in June 2021 [20].

Our characterization of the B.1.258 ∆H69/∆V70 variant that has been circulating in several European countries and appears to result in higher viral loads highlights the importance of vigilant genomic surveillance in properly identifying and tracking SARS-CoV-2 variants that display the potential to derail worldwide efforts to mitigate the pandemic.

## Supplementary Information

Below is the link to the electronic supplementary material.

Supplementary file 1 (PDF 51 kb)

## Data Availability

All sequences sequenced by the authors have been submitted to GISAID.
