# Supplementary material for: A SARS-CoV-2 mutant from B.1.258 lineage with ∆H69/∆V70 deletion in the Spike protein circulating in Central Europe in the fall 2020
Source: Virus Genes. 2021 Aug 27;57(6):556–60. doi: 10.1007/s11262-021-01866-5 (PMC8390540; doi:10.1007/s11262-021-01866-5)
Supplement: Supplementary file 1 — (PDF 51 kb) [file 11262_2021_1866_MOESM1_ESM.pdf]

GISAID Acknowledgements			
Originating lab	Submitting lab	Authors	Samples
St Vincent's Pathology (SydPath)	NSW Health Pathology - Institute of Clinical Pathology and Medical Research; Westmead Hospital; University of Sydney	CIDM-PH et al. et al	Australia/NSW1328/2020 (EPI_ISL_767894)
unknown	Public Health Virology Laboratory, Forensic and Scientific Services (PHV-FSS)	Son Nguyen et al. et al	Australia/QLD1140/2020 (EPI_ISL_593641)
Department of Clinical Microbiology	GIGA Medical Genomics	Keith Durkin et al	Belgium/ULG-10838/2020 (EPI_ISL_666873)
DB Diagnosticos do Brasil	Laboratório de Parasitologia Médica - Instituto de Medicina Tropical - Universidade de São Paulo	Nuno Faria et al	Brazil/AM-L70-CD1722/2020 (EPI_ISL_804823)
Laboratorio de Virologia Molecular / UFRJ	Bioinformatics Laboratory / LNCC	Carolina M Voloch et al	Brazil/RJ-00541/2020 (EPI_ISL_717925)
Genetica Molecular and Subdepartamento de Virologia ISP Chile	Instituto de Salud Publica de Chile	Javier Tognarelli et al	Chile/MA-194116-B/2020 (EPI_ISL_746529)
The National Institute of Public Health	State Veterinary Institute Prague	Nagy et al	CzechRepublic/NRL_10181/2020 (EPI_ISL_626625), CzechRepublic/NRL_10421/2020 (EPI_ISL_737012), CzechRepublic/NRL_10594/2020 (EPI_ISL_737032), CzechRepublic/NRL_11782-2/2020 (EPI_ISL_792704), CzechRepublic/NRL_8884/2020 (EPI_ISL_626577), CzechRepublic/NRL_9840/2020 (EPI_ISL_626609), CzechRepublic/NRL-9962/2020 (EPI_ISL_660554)

GISAID Acknowledgements			
Originating lab	Submitting lab	Authors	Samples
Department of Virus and Microbiological Special Diagnostics, Statens Serum Institut, Copenhagen, Denmark	Albertsen Lab, Department of Chemistry and Bioscience, Aalborg University, Denmark	Danish Covid-19 Genome Consortium et al	Denmark/DCGC-12314/2020 (EPI_ISL_682495), Denmark/DCGC-19132/2020 (EPI_ISL_748268), Denmark/DCGC-23126/2020 (EPI_ISL_793371), Denmark/DCGC-24852/2020 (EPI_ISL_795339), Denmark/DCGC-27186/2020 (EPI_ISL_817500)
Department of Virus and Microbiological Special Diagnostics, Statens Serum Institut, Denmark	Albertsen lab, Department of Chemistry and Bioscience, Aalborg University, Denmark	Danish Covid-19 Genome Consortia et al	Denmark/DCGC-1278/2020 (EPI_ISL_618071), Denmark/DCGC-2203/2020 (EPI_ISL_617504), Denmark/DCGC-4406/2020 (EPI_ISL_621827)
Respiratory Virus Unit, Microbiology Services Colindale, Public Health England	Respiratory Virus Unit, Microbiology Services Colindale, Public Health England	Monica Galiano et al	England/20130069504/2020 (EPI_ISL_423656)
Respiratory Virus Unit, National Infection Service, Public Health England	COVID-19 Genomics UK (COG-UK) Consortium	PHE Covid Sequencing Team et al	England/205082085/2020 (EPI_ISL_733592)
Lighthouse Lab in Alderley Park	Wellcome Sanger Institute for the COVID-19 Genomics UK (COG-UK) consortium	Jacquelyn Wynn et al	England/ALDP-A6F90A/2020 (EPI_ISL_607092)

GISAID Acknowledgements			
Originating lab	Submitting lab	Authors	Samples
Lighthouse Lab in Alderley Park	Wellcome Sanger Institute for the COVID-19 Genomics UK (COG-UK) Consortium	Jacquelyn Wynn et al	England/ALDP-B24B28/2020 (EPI_ISL_647005), England/ALDP-DC5EC4/2020 (EPI_ISL_822058), England/ALDP-DCB295/2020 (EPI_ISL_822172)
Lighthouse Lab in Cambridge	Wellcome Sanger Institute for the COVID-19 Genomics UK (COG-UK) Consortium	Rob Howes et al	England/CAMC-B1FC1E/2020 (EPI_ISL_647519)
Department of Pathology, University of Cambridge	COVID-19 Genomics UK (COG-UK) Consortium	Aminu S. Jahun et al	England/CAMC-BBB92C/2020 (EPI_ISL_704384)
Lighthouse Lab in Milton Keynes	Wellcome Sanger Institute for the COVID-19 Genomics UK (COG-UK) consortium	The Lighthouse Lab in Milton Keynes et al	England/MILK-9AB3C9/2020 (EPI_ISL_551056), England/MILK-A7DC1F/2020 (EPI_ISL_606346), England/MILK-AB86B0/2020 (EPI_ISL_623362)
Lighthouse Lab in Milton Keynes	Wellcome Sanger Institute for the COVID-19 Genomics UK (COG-UK) Consortium	The Lighthouse Lab in Milton Keynes et al	England/MILK-C2F60D/2020 (EPI_ISL_782957)
Quadram Institute Bioscience	COVID-19 Genomics UK (COG-UK) Consortium	Dave J. Baker et al	England/NORW-F2CFE/2020 (EPI_ISL_650120)
Lighthouse Lab in Glasgow	Wellcome Sanger Institute for the COVID-19 Genomics UK (COG-UK) consortium	Harper VanSteenhouse et al	England/QEUH-9611E3/2020 (EPI_ISL_531939), England/QEUH-96F75C/2020 (EPI_ISL_540238), Scotland/QEUH-9B85F0/2020 (EPI_ISL_568224)

GISAID Acknowledgements			
Originating lab	Submitting lab	Authors	Samples
Virology Department, Sheffield Teaching Hospitals NHS Foundation Trust/Department of Infection, Immunity and Cardiovascular Disease, The Medical School, University of Sheffield	COVID-19 Genomics UK (COG-UK) Consortium	Thushan de Silva et al	England/SHEF-CA66A/2020 (EPI_ISL_680168)
CNR Virus des Infections Respiratoires - France SUD	CNR Virus des Infections Respiratoires - France SUD	Antonin Bal et al	France/ARA-96089/2020 (EPI_ISL_732701)
CHU Purpan - Laboratoire de Virologie - Institut Fédératif de Biologie	CHU Purpan - Laboratoire de Virologie - Institut Fédératif de Biologie	Latour J. et al	France/OCC-189/2020 (EPI_ISL_804373)
University of Debrecen, Department of Medical Microbiology	National Laboratory of Virology, Szentágothai Research Centre	Endre Gábor Tóth et al	Hungary/UD-90512/2020 (EPI_ISL_671473)
The National University Hospital of Iceland	deCODE genetics	Daniel F Gudbjartsson et al	Iceland/352/2020 (EPI_ISL_424376)
CSIR-Indian Institute of Chemical Biology, MEDICA Supercpecialty Hospital Kolkata	CSIR-Indian Institute of Chemical Biology, MEDICA Supercpecialty Hospital Kolkata	Sujay Krishna Maity et al	India/WB-IICB-036/2020 (EPI_ISL_661309)
National Virus Reference Laboratory	National Virus Reference Laboratory	Michael Carr et al	Ireland/CO-NVRL-75IRL32904/2020 (EPI_ISL_671872), Ireland/KE-NVRL-73IRL40299/2020 (EPI_ISL_578300)
Pathogen Genomics Center, National Institute of Infectious Diseases	Pathogen Genomics Center, National Institute of Infectious Diseases	Tsuyoshi Sekizuka et al	Japan/IC-0489/2020 (EPI_ISL_768698)
Institute for Medical Research, Infectious Disease Research Centre, National Institutes of Health, Ministry of Health Malaysia	Institute for Medical Research, Infectious Disease Research Centre, National Institutes of Health, Ministry of Health Malaysia	Suppiah J et al	Malaysia/IMR-WI194/2020 (EPI_ISL_718307)
Ministry of Health Hospitals	Institute of Health and Community Medicine	David Perera et al	Malaysia/UNIMAS-M4061/2020 (EPI_ISL_718165)

GISAID Acknowledgements			
Originating lab	Submitting lab	Authors	Samples
Dutch COVID-19 response team	National Institute for Public Health and the Environment (RIVM)	Adam Meijer et al	Netherlands/GE-RIVM-20154/2020 (EPI_ISL_723167)
LabPLUS	Institute of Environmental Science and Research (ESR)	Xiaoyun Ren et al	NewZealand/20CV0692/2020 (EPI_ISL_755627)
Ostfold Hospital Trust - Kalnes, Centre for Laboratory Medicine, Section for gene technology and infection serology	Norwegian Institute of Public Health, Department of Virology	Kathrine Stene-Johansen et al	Norway/3715/2020 (EPI_ISL_590987)
Haukeland University Hospital, Dept. of Microbiology	Norwegian Institute of Public Health, Department of Virology	Kathrine Stene-Johansen et al	Norway/5852/2020 (EPI_ISL_775336)
Laboratory for Respiratory Viruses, Cantacuzino National Military-Medical Institute for Research and Development	Cantacuzino Institute	M.Lazar et al	Romania/284213/2020 (EPI_ISL_455468)
HELIX LLC	WHO National Influenza Centre Russian Federation	Andrey Komissarov et al	Russia/MOS-RII-MH9270S/2020 (EPI_ISL_733427), Russia/PSK-RII-MH6191S/2020 (EPI_ISL_733361)
Institute of Virology, Biomedical Research Center of the Slovak Academy of Sciences, Bratislava	Faculty of Natural Sciences, Comenius University, Bratislava	Viktória Hodorová et al	Slovakia/UKBA-403/2020 (EPI_ISL_718251)
Institute of Virology, Biomedical Research Center of the Slovak Academy of Sciences, Bratislava	Faculty of Natural Sciences, Comenius University, Bratislava	Kristína Boršová et al	Slovakia/UKBA-404/2020 (EPI_ISL_718252), Slovakia/UKBA-604/2020 (EPI_ISL_788982)
NHLS-IALCH	KRISP, KZN Research Innovation and Sequencing Platform	Giandhari J et al	SouthAfrica/KRISP-DG01842216/2020 (EPI_ISL_736947)
NHLS Universitas Academic	UFS Virology	PA Bester et al	SouthAfrica/UFS-VIRO-NGS-35/2020 (EPI_ISL_682342)

GISAID Acknowledgements			
Originating lab	Submitting lab	Authors	Samples
Division of Emerging Infectious Diseases, Bureau of Infectious Diseases Diagnosis Control, Korea Disease Control and Prevention Agency	Division of Emerging Infectious Diseases, Bureau of Infectious Diseases Diagnosis Control, Korea Disease Control and Prevention Agency	Ae Kyung Park et al	SouthKorea/KDCA0108/2020 (EPI_ISL_747344)
Klinsisk mikrobiologi Linkoping	The Public Health Agency of Sweden	Department of Microbiology et al	Sweden/20-53002/2020 (EPI_ISL_661285)
Viollier AG	Department of Biosystems Science and Engineering, ETH Zürich	Christian Beisel et al	Switzerland/BL-ETHZ-230022/2020 (EPI_ISL_516565), Switzerland/BL-ETHZ-270140/2020 (EPI_ISL_541451)
University Hospital Basel, Clinical Virology	University Hospital Basel, Clinical Bacteriology	Madlen Stange et al	Switzerland/BS-42376604/2020 (EPI_ISL_581933)
Viollier AG	Department of Biosystems Science and Engineering, ETH Zürich	Chaoran Chen et al	Switzerland/SG-ETHZ-430703/2020 (EPI_ISL_796464), Switzerland/ZH-ETHZ-410010/2020 (EPI_ISL_737518)
1-Laboratory of Microbiology, National Reference Lab, Charles Nicolle Hospital; 2-University of Tunis ElManar, Faculty of Medicine of Tunis, LR99ES09, Tunis, Tunisia	1-Clinical and Experimental Pharmacology Lab, LR16SP02, National Center of Pharmacovigilance, University of Tunis El Manar, Tunis, Tunisia. 2-Neurodegenerative diseases and psychiatric troubles, LR18SP03, Razi Hospital, University of Tunis El Manar, Tunis, Tunisia. 3-Ministry of Health, National Observatory of New and Emerging Diseases, 1006, Tunis, Tunisia	Ilhem Boutiba-Ben Boubaker et al	Tunisia/3942/2020 (EPI_ISL_699657), Tunisia/61627/2020 (EPI_ISL_707698)
Uganda Central Public Health Lab and Uganda Virus Research Institute	MRC/UVRI & LSHTM Uganda Research Unit	Matthew Cotten et al	Uganda/UG089/2020 (EPI_ISL_738000)

GISAID Acknowledgements			
Originating lab	Submitting lab	Authors	Samples
Helix/Illumina	Genomics and Discovery, Respiratory Viruses Branch, Division of Viral Diseases, Centers for Disease Control and Prevention	Peter W. Cook et al	USA/AL-CDC-STM-034/2020 (EPI_ISL_802654)
Massachusetts General Hospital	Infectious Disease Program, Broad Institute of Harvard and MIT	Lemieux et al	USA/MA-MGH-03435/2020 (EPI_ISL_765837)
Utah Public Health Laboratory	Utah Public Health Laboratory	Erin Young et al	USA/NH-UPHL-2012558654/2020 (EPI_ISL_738357)
Wadsworth Center, New York State Department.of Health	Wadsworth Center, New York State Department.of Health	Kirsten St. George et al	USA/NY-Wadsworth-222417-01/2020 (EPI_ISL_765509)
Houston Methodist Hospital	Houston Methodist Hospital	S. Wesley Long et al	USA/TX-HMH-5181/2020 (EPI_ISL_544580)
Wales Specialist Virology Centre Sequencing lab: Pathogen Genomics Unit	COVID-19 Genomics UK (COG-UK) Consortium	Catherine Moore et al	Wales/PHWC-16C03E/2020 (EPI_ISL_572878)
National Institute for Communicable Disease Control and Prevention (ICDC) Chinese Center for Disease Control and Prevention (China CDC)	National Institute for Communicable Disease Control and Prevention (ICDC) Chinese Center for Disease Control and Prevention (China CDC)	Zhang et al	Wuhan/Hu-1/2019 (EPI_ISL_402125)
